# Self-Management Experiences of Adolescents With Diabetes Mellitus After Participating in a Structured Diabetes Education Program: A Qualitative Systematic Review and Thematic Synthesis

**DOI:** 10.1177/26350106261422691

**Published:** 2026-02-26

**Authors:** Paniarepa Saard, Albert Farre, Jan R. Boehnke, Cari Malcolm

**Affiliations:** School of Health Sciences, University of Dundee, Dundee Scotland; Department of Child and Adolescent Nursing, Princess Agrarajakumari College of Nursing, Chulabhorn Royal Academy, Thailand; School of Health Sciences, University of Dundee, Dundee Scotland; School of Health Sciences, University of Dundee, Dundee Scotland; School of Health Sciences, University of Dundee, Dundee Scotland

## Abstract

**Purpose:**

The purpose of this review was to systematically identify, explore, and synthesize findings from primary qualitative studies on adolescents’ self-management experiences with type 1 diabetes (T1DM) and type 2 diabetes (T2DM) following participation in a structured diabetes education program.

**Methods:**

Systematic searches were conducted in CINAHL, MEDLINE, and PsycINFO without restrictions on publication dates or language. Any qualitative or mixed-methods studies reporting the self-management experience and/or perspectives of adolescents ages 10 to 24 with either T1DM or T2DM following participation in a structured diabetes education program were included. Tools from the Clinical Appraisal Skills Program were used to evaluate study quality, and thematic synthesis was employed to analyze the qualitative data.

**Results:**

Four studies focused on adolescents with T1DM were included, with no studies found addressing the experiences of adolescents with T2DM. Thematic synthesis revealed 6 analytical themes. Three themes related to experiences of T1DM self-management: (1) self-confidence in diabetes self-management, (2) improving diabetes self-management practice, and (3) coping with diabetes; three further themes related to the barriers and facilitators to T1DM self-management: (1) parents’ attitude and understanding of the condition, (2) peer support, and (3) communication with health care providers.

**Conclusion:**

Several factors were identified as valuable in helping adolescents with T1DM improve self-management adherence. However, evidence on self-management experiences after participating in a structured diabetes education program for adolescents with T2DM remains limited. There is urgent need for future research to find the best ways to support and empower young people in self-managing their diabetes through tailored education.

The incidence of both type 1 diabetes (T1DM) and type 2 diabetes (T2DM) has increased among individuals under the age of 20 over the past 2 decades.^1^ Recent estimates indicate that approximately 149 500 children and adolescents under the age of 20 would have been newly diagnosed with T1DM worldwide in 2021^2^ and 41 600 new cases of diagnosed T2DM.^3^ Diabetes is a condition that necessitates continued management. Insufficient regulation of blood glucose levels in adolescence can result in a range of complications developing in adulthood, including nephropathy, hypertension, neuropathy, and retinopathy.^4^

Research has indicated that young people often do not reach recommended glycemic targets, which is associated with poor metabolic control and rising levels of A1C.^5,6^ Irrespective of the specific type of diabetes and their different causal mechanisms, adolescents need to engage in a regimen of self-management behaviors to achieve good glycemic control. Self-management behaviors include lifestyle modifications, such as maintaining a healthy diet and body weight and engaging in regular physical activity, and taking medications as prescribed.^7,8^

Although parents support their children in practicing these behaviors, as they reach adolescence, they will be expected to become more autonomous.^9^ However, the physiological, psychological, and social changes that occur during adolescence can make it challenging for young people to engage in and maintain diabetes self-management behaviors.^10^ This could lead to a decline in glycemic stability in previously well-managing young people. Furthermore, engaging in self-management behaviors at school and adjusting the required routine of diabetes management, which often includes the addition of extra time and exercising, and keeping a balanced diet and well-directed relationships with friends, may make them feel frustrated and they may neglect following the diabetes regimen.^11^ Acknowledging the challenges in self-management among adults with diabetes compared to young people is important because these challenges can vary significantly due to factors such as lifestyle, work and family responsibilities, and health complications.^12^

Self-management in adolescents with T1DM requires the development of diabetes management skills focusing on insulin administration and glucose monitoring.^13,14^ Previous research has shown that adolescents with T1DM often struggle with insulin injection administration, blood glucose monitoring, meal plan adherence, and regular exercise.^15,16^ Adolescents with T1DM may lack confidence and feel uncertain about their abilities to independently make decisions regarding calculation of insulin doses and meal planning after completing diabetes education.^17,18^ Ongoing support from health care providers is necessary following completion of a diabetes program and is required to lead to sustained improvement in self-management behaviors.

Self-management in adolescents with T2DM focuses on a healthy lifestyle through behavioral changes in managing their diet and physical activity.^13,14^ Although adolescents may have learned how to manage their meal planning and regularly engage in physical activity through attending a diabetes education course, they can feel concerned about applying self-management practices in their daily lives post-program.^19-21^ For example, when eating out at the weekend or on a holiday, adolescents who do not manage routine lives with regular mealtimes can struggle to integrate self-management practice into their everyday lives and achieve stable blood glucose.^18,20,21^ Parent and peer support could help adolescents overcome the difficulties of managing meal plans related to insulin administration and provide reminders to check blood glucose or control their diet.^11,22^ Thus, providing age-appropriate education programs to assist adolescents in facing these challenges of self-management is one of the strategies for improving and maintaining diabetes self-management behaviors long-term.

“Structured education” refers to patient-centered group programs with a clear philosophy, documented curriculum, supportive resources, and a foundation in relevant learning and psychological theories.^23^ Effective education programs must meet 4 key criteria: They should be structured, have a written curriculum, be delivered by trained educators, and be quality assured.^24^ Structured diabetes education programs have been recommended and provided as a national guideline for the management of diabetes.^25^

The Child and Adolescent Structured Competencies Approach to Diabetes Education (CASCADE) is an example of a structured diabetes education program developed for children and adolescents with T1DM in the UK.^26^ This intervention, delivered by diabetes nurses and dietitians, employs psychological techniques that are solution-focused and motivational interviewing approaches to enhance engagement and behavior change in adolescents. However, a cluster-randomized controlled trial of the intervention did not report an improvement in A1C in adolescents at 12 or 24 months after completing the course.^26^ For T2DM, the diabetes self-management education (DSME) program was launched globally as a crucial aspect of care.^27^ This program is based on empowering patients with T2DM with knowledge, skills, and self-efficacy in order to inform their choices for diabetes self-management. A meta-analysis has shown DSME to improve glycemic control and demonstrated positive health outcomes in adults but indicated lower efficacy in children and adolescents in terms of glycemic control and psychosocial outcomes.^28^ The different life stages and challenges children and adolescents face compared to adults might contribute to the lower efficacy of DSME in children and adolescents.^28^

At the critical stage of transitioning from childhood to adulthood, adolescents with diabetes encounter unique difficulties inherent in achieving better diabetes self-management and sustaining their behaviors after completing diabetes education.^11,12^ Although various studies indicated that structured diabetes education programs may improve glycemic control (shown by reducing A1C), diabetes self-management, and positive health outcomes in both adults and adolescents with diabetes,^26,29-31^ adolescents’ experiences when approaching self-management and the maintenance of their behaviors following participation in structured diabetes education programs have not been extensively investigated. This systematic review and thematic synthesis therefore aimed to identify, explore, and synthesize findings from primary qualitative studies focused on the self-management experiences of adolescents with diabetes after participating in a structured diabetes education program. The key objectives were to (1) explore the experiences and views of adolescents concerning their diabetes self-management and (2) identify barriers and facilitators from adolescents’ experiences and views about diabetes self-management in continuing to implement their learning following participation in a structured diabetes education program. We asked the following research question:

What are the experiences and views of adolescents concerning their diabetes self-management after participating in a structured diabetes education program?

## Methods

The aim of this review was to identify an exhaustive body of research reporting on self-management experiences of adolescents with diabetes after participating in a structured diabetes education program and to synthesize qualitative findings from studies on similar phenomena to generate a deeper and more comprehensive understanding. The combination of a systematic review and thematic synthesis addresses these goals.^32^

This systematic review and thematic synthesis were conducted and reported in accordance with the Enhancing Transparency in Reporting the Synthesis of Qualitative Research (ENTREQ) statement checklist,^33^ and the Preferred Reporting Items for Systematic Reviews and Meta-Analyses (PRISMA) guidelines^34^ were used to guide reporting and illustrate the study selection process. The protocol of this study was registered with PROSPERO (No. CRD42022302727).

### Eligibility Criteria

The PICOS tool was used to formulate eligibility criteria for the systematic review. Criteria for inclusion included (1) (study design) primary research using qualitative methods or mixed-methods studies reporting qualitative data separately and (2) qualitative data reporting (outcomes) the experiences and/or views of (population) adolescents with T1DM or T2DM ages 10 to 24 years regarding diabetes self-management after participating in (intervention) a structured diabetes education program. The structure diabetes education programs reported must meet the following criteria^24^ : (1) have a structured written curriculum delivered in person, in writing, or online; (2) may or may not be delivered by trained educators; may be delivered individually, in groups, or mixed; and may include family/caregiver and (3) may involve a lower level of structural support, for example, allowing patients to access virtual clinics when required, which may include written materials, involve monitoring systems, and be assisted by technology. Studies reporting only on adolescents’ experiences and/or views regarding self-management before or during participating in a structured diabetes education program were excluded. For the inclusion and exclusion criteria used to select the studies, see Table 1.

**Table 1.** Inclusion and Exclusion Criteria

Criteria	Inclusion	Exclusion
Study design	Qualitative studies or mixed-method studies reporting adolescents’ experiences and/or views regarding diabetes self-management after participating in a structured diabetes education program	All quantitative studies Qualitative data reporting only on adolescents’ experiences and/or views regarding self-management before or during participating in a structured diabetes education program
Intervention	A structured diabetes education program, criteria include: have a structured written curriculum delivered in person, in writing, or online; may or may not be delivered by trained educators; may be delivered individually, in groups, or mixed; and may include family/caregiver; may involve a lower level of structural support, such as virtual clinics, written materials, monitoring systems, and be assisted by technology	
Population	The participants ages 10 y to 24 y and have type 1 diabetes or type 2 diabetes	Adults ages over 24 y and children ages under 10 y
Published date and language	No restriction applied concerning the year of publication or language	

### Search Strategy

A search was conducted on the Cochrane and PROSPERO databases to find completed or ongoing reviews on the subject, and none were identified. Searches were conducted on electronic databases MEDLINE, CINAHL, and PsycINFO, with no publication date and language restrictions. For the search terms applied, see Table 2; a sample search strategy for all databases is presented in Appendix 1 in the supplementary material.

**Table 2.** The Search Terms Used

S1 = adolescen* OR teen* OR teenager* OR youth OR “young people” OR “young adult” AND S2 = diabet* OR “diabetes mellitus” OR “type 1 diabetes” OR “type 2 diabetes” OR “Diabetes Mellitus, Type 1” OR “Diabetes Mellitus, Type 2” OR “T1D” OR “T2D” AND S3 = experience* OR perception* OR view* OR opinion* OR facilitator* OR barrier* OR S4 = qualitative OR interview* OR observation* OR focus group*

### Selection of Studies

Articles located in the searches were imported to the EndNote reference manager (version 20), and duplicates were removed. All titles and abstracts of the identified records were screened using Rayyan software^35^ based on the inclusion/exclusion criteria by the first reviewer (PS), and a sample of 10% (n = 560) was reviewed by a second reviewer (JB). The studies agreed by both reviewers for inclusion in full-text screening were then mixed together with a random sample of articles (n = 550) from the full data base for checking by a third reviewer (AF). The full texts of the remaining articles were retrieved and assessed for eligibility by the first (PS) and second (JB) reviewers. The third reviewer (AF) separately cross-checked the articles, and conflicts were resolved by consensus between all reviewers.

### Data Extraction

According to Cochrane’s guideline for data extraction of qualitative evidence for systematic reviews,^36^ a data extraction form was developed and used to collect important study characteristics and other relevant information, including author, title, year of publication, country, study objectives, study design, sample size, participants details, intervention, and findings (Appendix 2 in the supplementary material). The complete item extraction was checked by another reviewer (JB or AF).

### Quality Appraisal

The full texts of selected publications were assessed for quality by 2 independent reviewers (PS and JB) using the CASP (Critical Appraisal Skills Program) quality appraisal tool for qualitative studies.^37^ The CASP tool was selected to assess the quality of the included studies because it provides a methodological framework that allows for critical reflection on aspects to ensure transparency and consistency in evaluating studies for inclusion in qualitative syntheses. This made it well-suited for evaluating the diverse range of qualitative studies included in this meta-synthesis.

Any disagreements between reviewers were resolved in discussion with a third reviewer (AF). The overall quality assessment of included studies was performed by rating CASP items (Appendix 3 in the supplementary material). All of the included studies had a clear statement of the research objectives and appropriate qualitative methodology, and descriptions of data analysis techniques were generally provided.

### Data Synthesis

A thematic synthesis method^38^ was used to categorize and analyze the data from the included studies. NVivo software (release 1.0) was used to assist with the data analysis process. In the first stage of the process, PS read all the extracted data from included publications thoroughly and coded text line by line to generate initial codes. Then, all reviewers independently coded a subset of the data, identified and discussed themes, and agreed on the final descriptive themes. Finally, the review team explored the differences and similarities between the themes to determine the connections between them, merging them where needed, and subsequently creating analytical themes. Analytical themes were developed by “going beyond” the initial study findings and generating new interpretive constructs or explanations. Analytical themes were inferred based on the descriptive themes and the initial review question posed. The draft summary of the main themes was prepared and reviewed by the review team and refined until it was agreed on.

## Results

**Figure 1.**
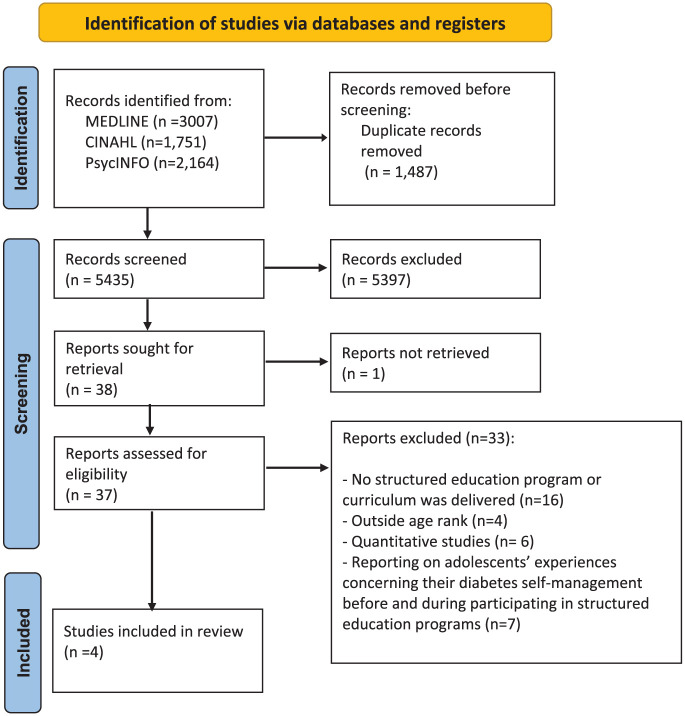
PRISMA 2020 flow diagram for new systematic reviews, which included searches of databases and registers only.^34^

A total of 5435 identified records were screened for eligibility against the inclusion criteria, resulting in 40 records considered eligible or inconclusive. One article was not retrieved because of a lack of access to the full text and not being available online or in library databases.^39^ The author of this article was contacted, but no response was received. Thirty-seven full-text articles were retrieved and assessed for eligibility. On screening the full texts, 16 articles were excluded because the studies did not deliver the intervention using a structured education program or curriculum, and 7 articles were excluded for reporting on adolescents’ experiences concerning their diabetes self-management before and during participating in structured education programs. Four articles were excluded because the participants were outside the age range, and 6 articles were excluded because they were quantitative studies. Four articles included in the final synthesis (Figure 1).

### Characteristics of the Included Studies

The four included studies were conducted in the United States,^40^ Denmark,^41^ England,^42^ and Australia^43^ between 2013 and 2021. Three studies delivered interventions face to face, and one study used only an online format via mobile phone apps. All studies employed a qualitative study with semi-structured interviews, observations, and focus groups for collecting the data. The four studies included data from 45 participants (36% male, 64% female). The participants were diagnosed with T1DM, and age ranged from 10 to 24 years. Three studies recruited participants from a diabetes clinic, and 1 study recruited from the community. Table 3 presents the study characteristics, and Table 4 outlines details of the interventions.

**Table 3.** Characteristics of Included Studies (N = 4)

Authors/year of publication	Country/setting	Study design	Sample size	Population	Intervention	Main finding according to authors
Sanders et al^42^ (2018)	UK Diabetes clinic	Qualitative study using semi-structured interviews, focus groups and observations	15	All White British Age: 16-24 y Diagnosed with type 1 diabetes for 9 y	WICKED (Working With Insulin, Carbohydrates, Ketones and Exercise to Manage Diabetes) Postintervention duration: 12 wk	Reported 3 themes: 1. We’re in it together 2. Tacit benefits 3. Transition beyond a structured education program
Shetty et al^43^ (2021)	Australia Diabetes clinic	Mixed method: semi-structured interviews and mobile app rating scale survey	10	All White Age: 12-24 y Diagnosed with type 1 diabetes >6 mo	A novel mHealth app acT1ve Postintervention duration: 6 wk	Reported 3 themes: 1. Increased knowledge (information) 2. Increased confidence to exercise (confidence) 3. Suitability for people who are less engaged in exercise
Ammentorp et al^41^ (2013)	Denmark Community	Qualitative study using semi-structured interviews	9	All White Age: 16-19 y Duration of type 1 diabetes: 9.7 y	Coaching program Postintervention duration: 1-3 mo	Reported 4 themes: 1. The experience of being met 2. Looking at myself and my diabetes in a new way 3. More self-esteem and energy 4. New tools to change routines
Hughes et al^40^ (2015)	USA Pediatric diabetes clinic	Qualitative focus group study	11	White (8), Black (1), Latino (2) Age: 9-12 y Duration of T1DM: 1-5 y	PREP-T1 (Preteen Re-Education With Parents-Type 1 Diabetes) Postintervention duration: not reported	Reported 3 themes: 1. Preteens’ perspectives on the value of interaction with a teen educator mentor 2. Learning with “CaBobby” (human patient simulation) the simulator 3. Learning topics for PREP-T1

**Table 4.** Characteristics of the Interventions

Name of the program	Goals and theory	Format and intervention	Teaching method	Duration
WICKED (Working With Insulin, Carbohydrates, Ketones and Exercise to Manage Diabetes) Sanders et al^42^ (2018)	- To build self-efficacy and new learning based on acknowledging the previous experiences young people have - Constructivist learning theory	- The course was delivered by diabetes specialist nurses and dietitians - Finishing with social issues on the final 2 d - 5 d session focuses on nutrition, insulin, and adjustment for daily life situations - 3 d for a recap of type 1 diabetes and carbohydrate-counting, progresses to key skills, such as sick-day rules and dealing with hypoglycemic episodes	- Written curriculum - Group discussion - One-to-one discussion with educators	5 d
MHealth app acT1ve Shetty et al^43^ (2021)	- The recommendation from International Society for Pediatric and Adolescent Diabetes: clinical practice consensus guidelines: exercise in children and adolescents with diabetes	- 6 wk for using the app to manage exercise twice a week - Data monitoring on exercise guidelines and advice and real-time support - Weekly contacts (technical issues, hypoglycemia)	- Mobile phone apps	6 wk
A coaching program Ammentorp et al^41^ (2013)	- To motivate the patients to take action, to make changes, and to achieve goals that were congruent with their life situation - Coaching method	- The course was delivered by professional certified coaches - 5 individual face-to-face coaching sessions (1.30 hr) - 3 telephone coaching sessions - Provide a proactive plan - The plan included different tools, such as “the wheel of life,” by which different aspects of life can be rated; templates for writing down barriers; resources; and values, goals, milestones, and action plans	- Written curriculum - Action plan - Face-to-face discussion	4 hr
PREP-T1 (Preteen Re-Education With Parents-Type 1 Diabetes feasibility intervention) Hughes et al^40^ (2015)	- To explore preteen-parent reeducation, collaborative decision-making, and peer support issues - Family management style framework	- The course was delivered by nurse diabetes educators - Providing interactive education based on human patient simulation - Group session with a teen educator mentor - Providing peer support and text, email, and/or phone conversation	- Written curriculum - Teen educator mentor-led discussion - Decision-making skills	1.30 hr

### Quality Appraisal

Using the CASP quality appraisal checklist, all studies were assessed as having a clear statement of aims, with an appropriate research design and methodology for addressing those aims. Most articles presented an appropriate recruitment strategy with data analysis that was judged to be sufficiently rigorous and provided a clear statement of findings (Appendix 3 in the supplementary material). Two articles^40,43^ were considered to be of high quality because they met all the CASP criteria, demonstrating clear methodological rigor, well-documented data collection and analysis processes, and evidence of ethical considerations. Two other articles^41,42^ were rated as medium quality due to a lack of information on ethical considerations and reflexivity.

### Thematic Synthesis Findings

Synthesis of findings from the 4 included studies resulted in the identification of 6 analytic themes that were inferred based on the descriptive themes. Three themes related to adolescents’ experiences of T1DM self-management after participating in a structured diabetes education program: (1) self-confidence in diabetes self-management, (2) improving diabetes self-management practice, and (3) coping with diabetes. Three further themes related to the barriers and facilitators to practicing T1DM self-management after participating in a structured diabetes education program: (1) parents’ attitude and understanding of the condition, (2) peer support, and (3) communication with health care providers. The descriptive themes that contributed to these analytical themes are outlined and presented in Table 5.

**Table 5.** Overview of Analytical Themes and Descriptive Themes

Objective	Analytical themes	Descriptive themes
To explore the experiences and views of adolescents concerning their diabetes self-management after participating in a structured diabetes education program	Self-confidence in diabetes self-management	Making decisions Feeling comfortable to change insulin doses Feeling more assured
	Improving diabetes self-management practice	Management of exercise Management of insulin administration Understanding optimal diabetes control
	Coping with diabetes	Struggling with diabetes Thinking positively with diabetes Feeling isolation
To identify barriers and facilitators from adolescents’ experiences and views about diabetes self-management in continuing to implement their learning after participating in a structured diabetes education program	Parents’ attitude and understanding of the condition	Parents’ understanding and assistance
	Peer support	Satisfaction with teen mentors Positive sharing experiences with peers
	Communication with health care providers	Clinic visit experiences Health care provider interaction

### Adolescents’ Experiences of T1DM self-Management After Participating in a Structured Diabetes Education Program

#### Self-confidence in diabetes self-management

This theme relates to the development of adolescents’ self-confidence in managing their metabolic control and other regimens in their daily lives and was identified in 3 out of the 4 studies.^40-43^ Twelve weeks following completion of a structured diabetes education program, adolescents described having the ability to make decisions about how to manage exercise intensity and duration, insulin administration, and other issues related to hypoglycemia.^42,43^ Being able to calculate and change insulin doses with confidence was also experienced by adolescents. They explained having an increased confidence to exercise and worrying less with experiencing a hypoglycemic episode while being physically active after immediately completing the program.^43^ Illustrative quotations are as follows:

Before I could not take my blood glucose at school, because I felt embarrassed that I had diabetes. I do not mind anymore. Now I just take it out in the middle of the class when that is what is needed.^41^

For me it wasn’t to try and learn too much about what diabetes is and stuff it was more giving me a chance to feel comfortable at kind of changing my insulin doses and being able to do it whilst I’m with people.^42^

I felt like I didn’t need to guess, and felt more assured in what I was doing to prepare for exercise; because information was coming from the app, it was probably going to work.^43^

#### Improving diabetes self-management practice

This theme illustrates the improvement of diabetes self-management practice from adolescents’ experiences, and this theme was identified in all 4 included studies.^40-43^ Adolescents expressed that they had more knowledge about engaging in skills to self-manage their diabetes and how to avoid serious diabetes complications. Skills learned during the diabetes education programs, such as how to count carbohydrates and measure blood glucose levels, were deemed to help adolescents in improving self-management practices. They also expressed that they “sought other learning experiences with the HPS [human patient simulation] such as hyperglycaemia, treating high blood glucose, counting carbohydrates, healthy eating and treating diabetes around participation in sports, more practice with the HPS, and practice with glucagon kits.”^40^

Illustrative quotations are as follows:

I think now we’ve been told how complex everything actually is with diabetes and how everything affects it I actually think about how dangerous it is to not check it. So, I do check it if that makes sense. Like I used to just oh you know it doesn’t really matter.^42^

The app would be particularly suitable for people who aren’t really into exercise yet or who have the fear of what happens if they go low during or after physical activity.^43^

It is especially the fact that I have changed my routines in the mornings. Now I measure my blood glucose and take insulin before I start working—in the dressing room.^41^

#### Coping with diabetes

This theme illustrates adolescents’ experiences after participating in the programs to cope with diabetes conditions and self-management, and this theme was identified in 2 of the 4 studies.^41,42^ Adolescents with T1DM felt relief and could cope with having the disease because they realized that in the coaching program, they were not the only ones who found it difficult to cope with having diabetes or to continue managing diabetes.^41^ They also explained how their attitude toward having diabetes has become more positive and how they were now more motivated to manage their condition.^42^ Illustrative quotations are as follows:

It has made me accept it more. For instance, I found it difﬁcult to tell people, that I had diabetes, but I feel that it has become much easier now.^41^

Everyone’s got their own story about diabetes. Some of it can be great, they can have you know perfect control, but I think nine times out of 10 most of us we really struggle with it.^42^

### Barriers and Facilitators to Practicing T1DM Self-Management After Participating in a Structured Diabetes Education Program

#### Parents’ attitude and understanding of the condition

This theme was identified in 2 of the 4 studies.^41,42^ Adolescents expressed that excessive control from parents was a negative attitude that diminished their motivation to self-manage their diabetes.^41^ They also described how parents having trust in their understanding of managing diabetes and maintaining a positive and motivating attitude is essential to support them to actively engage in self-management of their condition.^42^ Illustrative quotations are as follows:

In the long run I get sick and tired of mum and dad, when they keep saying: you must, you must now. . . . It is sort of negative, and then you meet such a positive attitude . . . and you too become more motivated in the long run.^41^

If I want to have a full fat coke which I don’t really do, but if I ever felt the need I could give insulin for it, I’ll be ﬁne. Like I can generally live a normal life. But my parents are like . . . if they see me eating a biscuit my mum like tuts cos, she doesn’t understand I can do this.^42^

#### Peer support

This theme was identified in 3 out of 4 studies.^40-42^ Adolescents explained how peer support services make them feel comfortable discussing their experiences and receiving support from others who face similar challenges, which encourages them to improve their diabetes self-management.^42^ Adolescents described how the support they received from teen educator mentors and peers during the program significantly enhanced their positive attitude toward diabetes self-management in the long term.^40^ Moreover, group discussions and problem-solving activities related to diabetes issues in the coaching program helped adolescents consider develop their individual thoughts and decision-making skills for managing their condition independently.^41^ Illustrative quotations are as follows:

You feel more comfortable speaking to people who have got the same issue as you . . . when you come into a group you feel more supported, and I think it gives you a bit of a boost up.^42^

Well, it is a whole different way of talking to people, they are sitting there following what you say, and instead of suggesting solutions on things, they are more likely to ask you for more information by asking different questions, so that you have to sit and think about what to do yourself like that.^41^

#### Communication with health care providers

As much as communicating with health care providers during clinic visits is important for supporting and giving the recommended treatment plan and diabetes management to adolescents, it also appears to be a potential barrier to their self-management. This theme was identified in 2 out of 4 studies.^41,42^ After completing the coaching program, adolescents reported that the support they received from their coach motivated them to improve their diabetes self-management. In contrast, interactions with health care staff, which adopted a more authoritative rather than empowering approach, made them feel unable to manage their condition effectively. Adolescents also expressed that during clinic visits, health care providers focused on the adolescents’ health condition, especially blood glucose levels, without considering the social and emotional obstacles to optimal glucose control. Illustrative quotations are as follows:

I think there’s when you come to clinic there’s a very . . . I wouldn’t say they explain it really, it’s a case of it’s always sort of drilled into you that you have your target range blood sugars, and you need to try and keep it in them.^42^

To be looked at in a different way. The staff just sees me as an impossible one. It is different with the coach . . . the coach makes me look at myself differently.^41^

## Discussion

This systematic review and thematic synthesis aimed to identify, explore, and synthesize findings from primary qualitative studies regarding the self-management experiences of adolescents with diabetes following participation in a structured diabetes education program. Four studies were included in this review, and an important descriptive finding of this review is that all studies were conducted with adolescents with T1DM. Overall, these findings suggest that more research is needed to enable a comprehensive understanding of adolescents’ experiences of diabetes self-management following participation in structured diabetes education programs. Equally, acknowledging the differences between T1DM and T2DM, it highlights a significant gap in the evidence and requirement to identify the self-management needs of adolescents with T2DM following participation in structured diabetes education programs.

This thematic synthesis identifies 6 analytical themes, describing changes in self-confidence in diabetes self-management, increased engagement in self-management practices, and improved coping with diabetes. The synthesis also highlights barriers and facilitators to practicing T1DM self-management after participating in a structured diabetes education program.

The first theme centers around self-confidence, which refers to individual strengths and abilities that help promote flexibility, demonstrating that patients are capable and effective in achieving their desired health outcomes. This is in line with findings from intervention studies,^44^ and may suggest that self-confidence can be maintained following participation in structured diabetes education programs: synthesis of the evidence suggests that developing self-confidence can motivate adolescents with T1DM to take action and change their behaviors to support adherence to self-management. This is comparable to the findings of this thematic synthesis, where adolescents with T1DM experienced increased self-confidence in managing exercise and insulin doses and less worry about having a hypoglycemic episode following participation in a structured diabetes education program.^42,43^ A previous study conducted by Eilander et al^45^ also indicated that low confidence in diabetes self-care and poorer self-management of diabetes among adolescents were both associated with higher A1C. Therefore, developing self-confidence is beneficial for adolescents to self-manage their condition because it contributes to motivation, treatment adherence, and self-care capabilities, ultimately leading to enhanced overall well-being and better glycemic control.

The second theme identified in this review, improving diabetes self-management practice, illustrated the value of gaining additional knowledge about their condition, which helped adolescents understand the importance of and adhere to diabetes management by integrating it into their lifestyle routines. Previous studies found that diabetes management knowledge enables adolescents with diabetes to practice autonomous self-management.^11,46,47^ This thematic synthesis reported that structured diabetes education programs provided through a mobile health application allows adolescents with T1DM to learn how to count carbohydrates, manage glucose levels while exercising, and contact health care providers via the app if they encounter issues with their management, helping to prevent hypoglycemia episodes.^43,48^

The third theme, coping with diabetes, refers to how to manage the challenges of diabetes in a positive way. Balancing self-management behaviors as a part of daily life was described as stressful by adolescents because they face difficulty trying to handle all of the complex aspects of their diabetes management in a busy social environment, such as at school.^49^ The findings from other research suggest that adolescents with T2DM view positive thinking as a key coping skill that can reduce the psychosocial burden associated with managing this chronic condition.^50,51^ Structured education programs can help ensure that coping skills for adolescents with T2DM are acquired more equitably. This thematic synthesis also illustrates the well-established finding that coping skills are essential for adhering to and continuing diabetes self-management in adolescents over the long-term.^41,42^ Coping skills are essential for diabetes self-management because they demonstrate that adolescents with diabetes who develop effective coping skills are better able to adhere to their self-management routines and maintain their management over the long-term.

Moving to the discussion of barriers and facilitators, the first analytic theme was parents’ attitude and understanding of the condition. Although adolescents are at a developmental stage where they are becoming more independent and responsible for their diabetes management, receiving positive support and encouragement from their parents is important to enhance better self-management and maintain good glycemic control.^52-54^ A previous study by Anderson et al^55^ showed that greater parental involvement in adolescents’ (ages 10-15 years) management of their T1DM is linked to improve metabolic control and that sharing responsibility for diabetes management tasks is associated with better self-care behavior in adolescents. Parental understanding of their child’s competence and motivation in carrying out self-management tasks and self-advocacy is linked to parent trust in their child’s ability to respond to their condition.^56^ In this systematic review, a similar finding indicates that parents’ understanding of their child’s diabetes plays a crucial role in facilitating adolescents’ more active self-management following program completion.^41,42^ Recent literature suggests that parents are better viewed as important assets who facilitate an incremental process toward self-care^57^ rather than as overly controlling, which can frustrate young people, create barriers to independence, and lead to conflict and resistance, resulting in reduced self-management behaviors.^49,58,59^ In the context of these findings, enabling parents to be more trusting and supporting adolescents’ autonomy in self-management activities could be a promising approach for DSME.

Peer support is another theme related to the barriers and facilitators that adolescents experience in their diabetes self-management. In the adolescent period, individuals’ social networks are readjusted such that they begin to favor their peers over support from members of the family.^60^ A previous study conducted by Lehmkuhl et al^61^ suggested that adolescents with T1DM would prefer additional diabetes management support, such as reminders to check their blood glucose or help with controlling their diet. However, for adolescents with diabetes, despite the strong support networks provided by peer groups, conflicts may arise due to the tension between the support they receive and the social pressure to fit in with their peers.^62^ Other studies have demonstrated the importance of peer support, particularly from those who have T1DM, and identified peer role models with good glycemic control as motivating influences.^63,64^ In addition, the presence of peers with the same condition was considered particularly beneficial because they can share their diabetes experiences with others in similar situations. This synthesis of evidence also highlighted that peer group discussions and sharing experiences of diabetes self-management through a structured education program were crucial components of the self-management experience even after the program ended.^40-42^ Although previous research suggests that peer support has a limited impact on immediate glycemic control,^65,66^ this thematic synthesis indicates that coaching and peer support are experienced as beneficial for fostering positive perspectives and motivation to self-manage their conditions.^40,41^ However, motivation and A1C reduction often decline in the months after the program, revealing a significant sustainability gap.^41^ Thus, implementing booster sessions or extended interventions that integrate peer support can help young people mitigate the isolation and diabetes burnout during their transition to independence.^67^

The final theme is communication with health care providers. The findings from this review illustrated interactions between adolescents and health care providers during clinic appointments. Health care providers mainly focused on optimal glucose control rather than emotional support to help adolescents overcome obstacles to achieving a range of blood glucose levels.^41,42^ Previous studies also revealed that health care providers often overlooked patients’ emotional well-being during routine clinic follow-up.^68,69^ Therefore, the lack of psychosocial support from health care providers could be a potential barrier to diabetes self-management in adolescents in the long-term.^56^

### Limitations

There are some limitations to this systematic review and thematic synthesis. First, the review identifies very few studies focusing on this topic. Although this reduces the robustness of the findings, this is a finding in itself, as is the fact that there were no studies investigating this topic from the perspective of adolescents living with T2DM. This is significant given the prevalence of T2DM is increasing in young people, and ensuring they have knowledge and understanding of required lifestyle modifications and glycemic control is paramount to prevent long-term complications and poor health outcomes.^13,14^ Second, all qualitative studies identified in the systematic search and included for thematic synthesis were conducted in Western countries and high-income countries. Moreover, most of the participants came from White ethnic groups. The findings may not be generalizable to other populations and stress the importance of undertaking further research that includes participants with wider demographic and multicultural characteristics. Third, the timing of the educational intervention could be considered as a potential limitation. If the program is offered only once, shortly after diagnosis, it may not adequately address the longer-term and evolving needs of young people in managing their diabetes.

Nevertheless, although only four qualitative studies met the inclusion criteria, each contributed rich and in-depth insights into adolescents’ self-management experiences with diabetes following a structured diabetes education program. The limited number of studies reflects both the specificity of the research focus and a broader gap in the literature, particularly the lack of research on T2DM. The synthesis of findings from these studies provides meaningful understanding and highlights consistent patterns across contexts. However, the small number of studies underscores the need to interpret the findings with caution, considering the potential limitations in generalizability, and points to the importance of further research to strengthen the evidence base.

## Conclusions

This thematic synthesis identified psychological and social support systems that mattered to the experiences of adolescents living with T1DM following their participation in a structured education program. For each of these support systems, previous research indicates some evidence of how these experiences could be leveraged to develop or adapt (such) interventions. However, there were no findings to address the self-management experiences of adolescents with T2DM after participating in a structured education program. With the increasing prevalence of T2DM in adolescents, the differences in treatment priorities and initial education between the 2 types of diabetes may lead to unique challenges in managing the condition. Further research is urgently needed to identify the best approaches for supporting and empowering young people in managing their diabetes through tailored education.

## Supplemental Material

sj-docx-1-tde-10.1177_26350106261422691 – Supplemental material for Self-Management Experiences of Adolescents With Diabetes Mellitus After Participating in a Structured Diabetes Education Program: A Qualitative Systematic Review and Thematic Synthesis Supplemental material, sj-docx-1-tde-10.1177_26350106261422691 for Self-Management Experiences of Adolescents With Diabetes Mellitus After Participating in a Structured Diabetes Education Program: A Qualitative Systematic Review and Thematic Synthesis by Paniarepa Saard, Albert Farre, Jan R. Boehnke and Cari Malcolm in The Science of Diabetes Self-Management and Care

sj-docx-2-tde-10.1177_26350106261422691 – Supplemental material for Self-Management Experiences of Adolescents With Diabetes Mellitus After Participating in a Structured Diabetes Education Program: A Qualitative Systematic Review and Thematic Synthesis Supplemental material, sj-docx-2-tde-10.1177_26350106261422691 for Self-Management Experiences of Adolescents With Diabetes Mellitus After Participating in a Structured Diabetes Education Program: A Qualitative Systematic Review and Thematic Synthesis by Paniarepa Saard, Albert Farre, Jan R. Boehnke and Cari Malcolm in The Science of Diabetes Self-Management and Care

sj-docx-3-tde-10.1177_26350106261422691 – Supplemental material for Self-Management Experiences of Adolescents With Diabetes Mellitus After Participating in a Structured Diabetes Education Program: A Qualitative Systematic Review and Thematic Synthesis Supplemental material, sj-docx-3-tde-10.1177_26350106261422691 for Self-Management Experiences of Adolescents With Diabetes Mellitus After Participating in a Structured Diabetes Education Program: A Qualitative Systematic Review and Thematic Synthesis by Paniarepa Saard, Albert Farre, Jan R. Boehnke and Cari Malcolm in The Science of Diabetes Self-Management and Care

sj-docx-4-tde-10.1177_26350106261422691 – Supplemental material for Self-Management Experiences of Adolescents With Diabetes Mellitus After Participating in a Structured Diabetes Education Program: A Qualitative Systematic Review and Thematic Synthesis Supplemental material, sj-docx-4-tde-10.1177_26350106261422691 for Self-Management Experiences of Adolescents With Diabetes Mellitus After Participating in a Structured Diabetes Education Program: A Qualitative Systematic Review and Thematic Synthesis by Paniarepa Saard, Albert Farre, Jan R. Boehnke and Cari Malcolm in The Science of Diabetes Self-Management and Care
