# Supplementary material for: Self-Management Experiences of Adolescents With Diabetes Mellitus After Participating in a Structured Diabetes Education Program: A Qualitative Systematic Review and Thematic Synthesis
Source: Sci Diabetes Self Manag Care. 2026 Feb 26;52(2):174–89. doi: 10.1177/26350106261422691 (PMC12996374; doi:10.1177/26350106261422691)
Supplement: sj-docx-1-tde-10.1177_26350106261422691 – Supplemental material for Self-Management Experiences of Adolescents With Diabetes Mellitus After Participating in a Structured Diabetes Education Program: A Qualitative Systematic Review and Thematic Synthesis [file sj-docx-1-tde-10.1177_26350106261422691.docx]

**Appendix 1: A search strategy for Medline database.**

| **EBSCOhost Search Databases**  **Database: MEDLINE**  **Search options:** Expanders - Apply related words; Apply equivalent subjects  Search modes - Boolean/Phrase | | |
| --- | --- | --- |
| **Search ID#** | **Search term** | **Results** |
| S1 | AB ( adolescen* OR teen* OR teenager* OR youth OR “young people”  OR “young adult” ) OR TI ( adolescen* OR teen* OR teenager* OR youth OR  “young people” OR “young adult” ) | (425,252) |
| S2 | AB ( diabet* OR “diabetes mellitus” OR “type 1 diabetes” OR “type 2 diabetes” OR “Diabetes Mellitus, Type 1” OR “Diabetes Mellitus, Type 2” OR “T1D” OR “T2D” )  OR TI ( diabet* OR “diabetes mellitus” OR “type 1 diabetes” OR “type 2 diabetes” OR “Diabetes Mellitus, Type 1” OR “Diabetes Mellitus, Type 2” OR “T1D” OR “T2D” ) | (699,523) |
| S3 | AB ( experience* OR perception* OR view* OR opinion* OR facilitator* OR barrier* ) OR TI ( experience* OR perception* OR view* OR opinion* OR facilitator* OR barrier* ) | (2,263,322) |
| S4 | AB ( qualitative OR interview* OR observation* OR focus group* ) OR TI  ( qualitative OR interview* OR observation* OR focus group* ) | (1,507,114) |
| S5 | S3 OR S4 | (3,468,625) |
| S6 | S1 AND S2 AND S5 | 3,007 |

**A search strategy for CINAHL database.**

| **EBSCOhost Search Databases**  **Database: CINAHL**  **Search options:** Expanders - Apply related words; Apply equivalent subjects  Search modes - Boolean/Phrase | | |
| --- | --- | --- |
| **Search ID#** | **Search term** | **Results** |
| S1 | I ( adolescen* OR teen* OR teenager* OR youth OR “young people”  OR “young adult” ) OR AB ( adolescen* OR teen* OR teenager* OR youth OR  “young people” OR “young adult” ) | (223,092) |
| S2 | I ( diabet* OR “diabetes mellitus” OR “type 1 diabetes” OR “type 2 diabetes” OR “Diabetes Mellitus, Type 1” OR “Diabetes Mellitus, Type 2” OR “T1D” OR “T2D” )  OR AB ( diabet* OR “diabetes mellitus” OR “type 1 diabetes” OR “type 2 diabetes” OR “Diabetes Mellitus, Type 1” OR “Diabetes Mellitus, Type 2” OR “T1D” OR “T2D” ) | (226,953) |
| S3 | I ( experience* OR perception* OR view* OR opinion* OR facilitator* OR barrier* ) OR AB ( experience* OR perception* OR view* OR opinion* OR facilitator* OR barrier* ) | (794,469) |
| S4 | I ( qualitative OR interview* OR observation* OR focus group* ) OR AB  ( qualitative OR interview* OR observation* OR focus group* ) | (479,793) |
| S5 | S3 OR S4 | (1,090,919) |
| S6 | S1 AND S2 AND S5 | 1,751 |

**A search strategy for PsycINFO database.**

| **#** | **Query** | **Results from 14 Feb 2022** |
| --- | --- | --- |
| 1 | (adolescen* or teen* or teenager* or youth or "young people" or "young adult").mp. [mp=title, abstract, heading word, table of contents, key concepts, original title, tests & measures, mesh word] | 644,987 |
| 2 | (diabet* or "diabetes mellitus" or "type 1 diabetes" or "type 2 diabetes" or "Diabetes Mellitus, Type 1" or "Diabetes Mellitus, Type 2" or "T1D" or "T2D").mp. [mp=title, abstract, heading word, table of contents, key concepts, original title, tests & measures, mesh word] | 35,586 |
| 3 | (experience* or perception* or view* or opinion* or facilitator* or barrier* or qualitative or interview* or observation* or focus group*).mp. [mp=title, abstract, heading word, table of contents, key concepts, original title, tests & measures, mesh word] | 1,919,695 |
| 4 | 1 and 2 and 3 | 2,164 |
