# Supplementary material for: Self-Management Experiences of Adolescents With Diabetes Mellitus After Participating in a Structured Diabetes Education Program: A Qualitative Systematic Review and Thematic Synthesis
Source: Sci Diabetes Self Manag Care. 2026 Feb 26;52(2):174–89. doi: 10.1177/26350106261422691 (PMC12996374; doi:10.1177/26350106261422691)
Supplement: sj-docx-2-tde-10.1177_26350106261422691 – Supplemental material for Self-Management Experiences of Adolescents With Diabetes Mellitus After Participating in a Structured Diabetes Education Program: A Qualitative Systematic Review and Thematic Synthesis [file sj-docx-2-tde-10.1177_26350106261422691.docx]

**Appendix 2:** **Common features of standardized data extraction forms.**

| **Data extraction field** | **Information extracted** |
| --- | --- |
| *Context and participants* | Detailed information is extracted on the study setting, participants, the intervention delivered etc. This may aid later interpretation and synthesis by helping to retain the context in which the data are embedded. |
| *Study design and methods used* | This includes the methodological approach taken by the study; the specific data collection and analysis methods utilized; and any theoretical models used to interpret or contextualize the findings. The data extraction approach, and therefore the data extraction template, may need to be flexible so as to accommodate data collected within different qualitative methodologies (ethnography, phenomenology etc.) and using different methods (interview, focus groups, observations, document analysis etc.). |
| *Findings* | This covers the key themes or concepts identified in the primary studies. In extracting these findings, some review authors attempt to distinguish between first and second order interpretations. |
| *Quality of the study* | Different approaches to appraising study quality have been used. |
