# Supplementary material for: Self-Management Experiences of Adolescents With Diabetes Mellitus After Participating in a Structured Diabetes Education Program: A Qualitative Systematic Review and Thematic Synthesis
Source: Sci Diabetes Self Manag Care. 2026 Feb 26;52(2):174–89. doi: 10.1177/26350106261422691 (PMC12996374; doi:10.1177/26350106261422691)
Supplement: sj-docx-3-tde-10.1177_26350106261422691 – Supplemental material for Self-Management Experiences of Adolescents With Diabetes Mellitus After Participating in a Structured Diabetes Education Program: A Qualitative Systematic Review and Thematic Synthesis [file sj-docx-3-tde-10.1177_26350106261422691.docx]

**Appendix 3: CASP quality appraisal scoresheet**

| **Study reference** | **Clear statement of aims?** | **Qualitative methodology appropriate?** | **Research design appropriate?** | **Recruitment strategy appropriate?** | **Data collection approach appropriate?** | **Researcher-participant relationship considered?** | **Ethical issues considered?** | **Data analysis sufficiently rigorous?** | **Clear statement of findings?** | **How valuable is this research?** | **Total scores** |
| --- | --- | --- | --- | --- | --- | --- | --- | --- | --- | --- | --- |
| Ammentorp et al^42^ | Y | Y | Y | Y | Y | Y | N | Y | Y | Y | M |
| Hughes et al^41^ | Y | Y | Y | Y | Y | Y | Y | Y | Y | Y | H |
| Sanders et al^43^ | Y | Y | Y | Y | Y | Y | N | Y | Y | Y | M |
| Shetty et al^44^ | Y | Y | Y | Y | Y | Y | Y | Y | Y | Y | H |

*Scoring: Y=Yes; CT=Cannot tell; G=High; M=Medium; L=Low*

Critical Appraisal Skills Programme (CASP) questions scoring: Yes =2 ? (Can’t Tell) = 1 No = 0

Total score 20 = high quality; 16–19 moderate quality; ≤ 15 low quality
